# Dichlorido(furfuryl­amine-κ*N*)(η^6^-hexa­methyl­benzene)­ruthenium(II)

**DOI:** 10.1107/S1600536811043170

**Published:** 2011-10-22

**Authors:** Amine Garci, Trieu-Tien Thai, Georg Süss-Fink, Bruno Therrien

**Affiliations:** a Institut de Chimie, Université de Neuchâtel, Avenue de Bellevaux 51, CH-2000 Neuchâtel, Switzerland

## Abstract

The single-crystal X-ray structure analysis of [RuCl_2_(C_12_H_18_)(C_5_H_7_NO)] reveals a distorted piano-stool geometry around the Ru^II^ atom, with a hexa­methyl­benzene ligand, two chloride ligands and a furfuryl­amine ligand, the latter coordinating through the amine group. In the crystal, a dimeric structure is observed as a result of N—H⋯Cl inter­actions between two symmetry-related mol­ecules.

## Related literature

For publications dealing with metal complexes of furfuryl­amine derivatives, see: Hu *et al.* (2006 ▶); Joesten *et al.* (1967 ▶). For reviews on arene–ruthenium complexes as anti­cancer agents, see: Süss-Fink (2010 ▶); Therrien & Smith (2011 ▶). For biological activity of metal complexes of furfuryl derivatives, see: Hamann *et al.* (1968 ▶); Shi *et al.* (2008 ▶). For a review on arene–ruthenium chemistry, see: Therrien (2009 ▶). For the synthesis, see: Bennett *et al.* (1982 ▶). For related structures, see: Govindaswamy *et al.* (2004 ▶); Therrien & Süss-Fink (2004 ▶); Therrien *et al.* (2004 ▶).
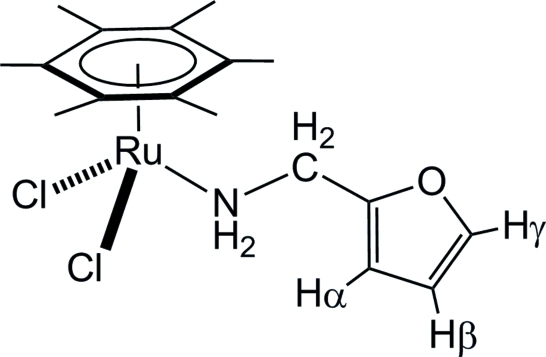


## Experimental

### 

#### Crystal data

[RuCl_2_(C_12_H_18_)(C_5_H_7_NO)]
*M* *_r_* = 431.35
Monoclinic, 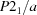

*a* = 7.6883 (6) Å
*b* = 22.8748 (18) Å
*c* = 10.1000 (7) Å
β = 100.493 (9)°
*V* = 1746.6 (2) Å^3^
*Z* = 4
Mo *K*α radiation
μ = 1.20 mm^−1^
*T* = 173 K
0.15 × 0.12 × 0.11 mm

#### Data collection

Bruker SMART CCD diffractometer
13771 measured reflections
3428 independent reflections
2693 reflections with *I* > 2σ(*I*)
*R*_int_ = 0.040

#### Refinement

*R*[*F* ^2^ > 2σ(*F* ^2^)] = 0.033
*wR*(*F* ^2^) = 0.081
*S* = 0.94
3428 reflections
205 parameters
H-atom parameters constrained
Δρ_max_ = 0.65 e Å^−3^
Δρ_min_ = −0.98 e Å^−3^

### 

Data collection: *SMART* (Bruker, 1999 ▶); cell refinement: *SMART* and *SAINT* (Bruker, 1999 ▶); data reduction: *SAINT*; program(s) used to solve structure: *SIR97* (Altomare *et al.*, 1999 ▶); program(s) used to refine structure: *SHELXTL* (Sheldrick, 2008 ▶); molecular graphics: *SHELXTL*; software used to prepare material for publication: *SHELXTL*.

## Supplementary Material

Crystal structure: contains datablock(s) I, global. DOI: 10.1107/S1600536811043170/ff2034sup1.cif

Structure factors: contains datablock(s) I. DOI: 10.1107/S1600536811043170/ff2034Isup2.hkl

Additional supplementary materials:  crystallographic information; 3D view; checkCIF report

## Figures and Tables

**Table 1:** Hydrogen-bond geometry (Å, °)

*D*—H⋯*A*	*D*—H	H⋯*A*	*D*⋯*A*	*D*—H⋯*A*
N—H1*A*⋯Cl2^i^	0.9	2.52	3.406 (3)	168

Symmetry code: (i) 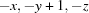.

**Fig. 1.**
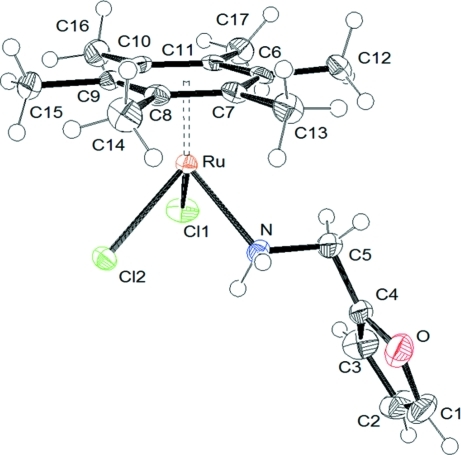
The molecular structure of (η6-C12H18)RuCl2(C5H7NO-κN). Displacement ellipsoids are drawn at the 50% probability level.

## supplementary crystallographic information

### Comment

Arene ruthenium(II) complexes are promising antitumoral and antimetastatic agents (Süss-Fink, 2010; Therrien & Smith, 2011), and furfuryl derivatives are known to possess antimetabolite (Hamann *et al.*, 1968) and antibacterial properties (Shi *et al.*, 2008). The synthesis of dichlorido(furfurylamine-κ*N*)(η^6^-hexamethylbenzene)ruthenium(II) is presented. However, a biological evaluation was not possible due to the low water-solubility of the compound.

The formation of (η^6^-C_12_H_18_)RuCl_2_(C_5_H_7_NO-κ*N*) is easily monitored by ^1^H NMR spectroscopy, in which the signal corresponding to the protons of the NH_2_ group is strongly shifted by 1.57 p.p.m., while the signal of the CH_2_ protons is also shifted downfield but to a lesser extent (0.15 p.p.m.) as compared to uncoordinated furfurylamine. The infrared spectrum of (η^6^-C_12_H_18_)RuCl_2_(C_5_H_7_NO-κ*N*) shows as well shifting of some of the bands associated to the furfurylamine moiety, especially those corresponding to the symmetrical and asymmetrical ν_NH_, in accordance with other metal complexes of furfurylamine derivatives (Joesten *et al.*, 1967). In addition, the molecular structure of the complex has been established by single-crystal structure analysis.

The title complex shows a three-legged piano-stool geometry with the Ru^II^ metal center being surrounded by an hexamethylbenzene ligand, two terminal chlorido and a *N*-coordinated furfurylamine ligand, see Fig. 1. The furfurylamine acts as a monodentate ligand and the Ru—N distance at 2.156 (2) Å is comparable to the one found in the analogous dichlorido(η^6^-*p*-cymene)(benzylamine-κ*N*)ruthenium(II) complex [2.1445 (18) Å] (Govindaswamy *et al.*, 2004). The aromatic ring of the hexamethylbenzene is planar and the Ru-centroid distance is 1.672 Å (centroid defined by C6 to C11). Otherwise, the Ru—Cl distances are almost equivalent at 2.4277 (9) and 2.4299 (8) Å, respectively, which is similar to those found in other dichlorido arene ruthenium complexes (Therrien & Süss-Fink, 2004; Govindaswamy *et al.*, 2004; Therrien *et al.*, 2004).

In the crystal packing, one chlorido is involved in an H-bonded interaction with the NH_2_ moiety of a neighbouring molecule, thus forming a centrosymmetric dimeric structure: The N—Cl separations being 3.406 (3)Å with the N—H···Cl angles being 168.3°.

### Experimental

Furfurylamine was purchased from Aldrich and used as received and [(η^6^-C_12_H_18_)Ru(µ-Cl)Cl]_2_ (Bennett *et al.*, 1982) was prepared according to published methods. The NMR spectrum was recorded on a Bruker 400 MHz spectrometer. The infrared spectrum was recorded on a Perkin-Elmer 1720X FT—IR spectrometer (4000–400 cm^-1^).

A mixture of [(η^6^-C_12_H_18_)Ru(µ-Cl)Cl]_2_ (90 mg, 0.135 mmol) and two equivalents of furfurylamine (24 µ*L*, 0.27 mmol) was stirred in dichloromethane (10 ml) for 2 h at room temperature. The orange-red compound which formed was filtered, washed with diethyl ether and dried under vacuum (Yield 98%).

Crystals suitable for X-ray diffraction analysis were obtained, after days, by slow diffusion of diethyl ether into a dichloromethane solution of the title complex.

^1^H NMR (400 MHz, CDCl_3_): 7.35 p.p.m. (d, ^3^*J* = 2 Hz, 1H, H_γ_), 6.29 (dd, 1H, H_β_), 6.20 (d, ^3^*J* = 2 Hz, 1H, H_α_), 3.96–3.92 (m, 2H, CH_2_), 2.97 (br, 2H, NH_2_), 2.15 (s, 18H, CH_3_).

IR (KBr pellet): ν_NHasym_ 3295 s, ν_NHsym_ 3194 m, ν_COC_ 1159 m, ν_COC_ 1000 m cm^-1^.

### Refinement

All H atoms were included in calculated positions (C—H = 0.93 Å for C_arom_, 0.97 Å for CH_2_, 0.96 Å for CH_3_; N—H = 0.90 Å for NH_2_) and treated as riding atoms with the constraint *U*_iso_(H) = 1.2 (1.5 for methyl) *U*_eq_(carrier) applied.

### Crystal data

**Table:**

[RuCl_2_(C_12_H_18_)(C_5_H_7_NO)]	*F*(000) = 880
*M**_r_* = 431.35	*D*_x_ = 1.640 Mg m^−^^3^
Monoclinic, *P*2_1_/*a*	Mo *K*α radiation, λ = 0.71073 Å
Hall symbol: -P 2yab	Cell parameters from 8000 reflections
*a* = 7.6883 (6) Å	θ = 2.0–26.1°
*b* = 22.8748 (18) Å	µ = 1.20 mm^−^^1^
*c* = 10.1000 (7) Å	*T* = 173 K
β = 100.493 (9)°	Block, orange
*V* = 1746.6 (2) Å^3^	0.15 × 0.12 × 0.11 mm
*Z* = 4	

### Data collection

**Table:**

Bruker SMART CCD PLATFORM diffractometer	2693 reflections with *I* > 2σ(*I*)
Radiation source: fine-focus sealed tube	*R*_int_ = 0.040
graphite	θ_max_ = 26.0°, θ_min_ = 2.1°
Detector resolution: 0 pixels mm^-1^	*h* = −9→9
ω scans	*k* = −28→28
13771 measured reflections	*l* = −12→12
3428 independent reflections	

### Refinement

**Table:**

Refinement on *F*^2^	Primary atom site location: structure-invariant direct methods
Least-squares matrix: full	Secondary atom site location: difference Fourier map
*R*[*F*^2^ > 2σ(*F*^2^)] = 0.033	Hydrogen site location: inferred from neighbouring sites
*wR*(*F*^2^) = 0.081	H-atom parameters constrained
*S* = 0.94	*w* = 1/[σ^2^(*F*_o_^2^) + (0.0567*P*)^2^] where *P* = (*F*_o_^2^ + 2*F*_c_^2^)/3
3428 reflections	(Δ/σ)_max_ = 0.002
205 parameters	Δρ_max_ = 0.65 e Å^−^^3^
0 restraints	Δρ_min_ = −0.98 e Å^−^^3^

### Special details

**Table:**

Experimental. A crystal was mounted at 173 K on a Bruker *SMART* CCD PLATFORM using Mo *K*α graphite monochromated radiation. Image plate distance 70 mm, φ oscillation scans 0 - 200°, step Δφ = 1.0°, 3 minutes per frame.
Geometry. All e.s.d.'s (except the e.s.d. in the dihedral angle between two l.s. planes) are estimated using the full covariance matrix. The cell e.s.d.'s are taken into account individually in the estimation of e.s.d.'s in distances, angles and torsion angles; correlations between e.s.d.'s in cell parameters are only used when they are defined by crystal symmetry. An approximate (isotropic) treatment of cell e.s.d.'s is used for estimating e.s.d.'s involving l.s. planes.
Refinement. Refinement of *F*^2^ against ALL reflections. The weighted *R*-factor *wR* and goodness of fit *S* are based on *F*^2^, conventional *R*-factors *R* are based on *F*, with *F* set to zero for negative *F*^2^. The threshold expression of *F*^2^ > σ(*F*^2^) is used only for calculating *R*-factors(gt) *etc*. and is not relevant to the choice of reflections for refinement. *R*-factors based on *F*^2^ are statistically about twice as large as those based on *F*, and *R*- factors based on ALL data will be even larger.

### Fractional atomic coordinates and isotropic or equivalent isotropic displacement parameters (Å^2^)

**Table:**

	*x*	*y*	*z*	*U*_iso_*/*U*_eq_	
C1	−0.2917 (6)	0.67556 (18)	−0.3554 (4)	0.0458 (11)	
H1	−0.3165	0.6690	−0.4478	0.055*	
C2	−0.3965 (6)	0.7033 (2)	−0.2866 (5)	0.0554 (14)	
H2	−0.5055	0.7201	−0.3215	0.067*	
C3	−0.3115 (6)	0.70300 (18)	−0.1486 (4)	0.0416 (10)	
H3	−0.3544	0.7188	−0.0760	0.050*	
C4	−0.1575 (5)	0.67527 (14)	−0.1450 (3)	0.0258 (7)	
C5	−0.0108 (5)	0.66154 (16)	−0.0330 (4)	0.0318 (8)	
H5A	0.0999	0.6643	−0.0657	0.038*	
H5B	−0.0086	0.6908	0.0368	0.038*	
C6	0.3433 (4)	0.64157 (14)	0.2398 (3)	0.0189 (7)	
C7	0.3751 (4)	0.58374 (14)	0.1941 (3)	0.0204 (7)	
C8	0.3450 (4)	0.53442 (14)	0.2709 (3)	0.0194 (7)	
C9	0.2821 (4)	0.54158 (14)	0.3960 (3)	0.0200 (7)	
C10	0.2485 (4)	0.59829 (14)	0.4412 (3)	0.0190 (7)	
C11	0.2777 (4)	0.64832 (14)	0.3620 (3)	0.0194 (7)	
C12	0.3872 (5)	0.69403 (15)	0.1635 (4)	0.0276 (8)	
H12A	0.3169	0.7266	0.1823	0.041*	
H12B	0.3624	0.6858	0.0687	0.041*	
H12C	0.5103	0.7033	0.1906	0.041*	
C13	0.4423 (5)	0.57521 (16)	0.0645 (3)	0.0281 (8)	
H13A	0.5586	0.5584	0.0834	0.042*	
H13B	0.4469	0.6123	0.0207	0.042*	
H13C	0.3641	0.5495	0.0066	0.042*	
C14	0.3796 (5)	0.47449 (15)	0.2214 (4)	0.0324 (8)	
H14A	0.3347	0.4720	0.1264	0.049*	
H14B	0.3218	0.4459	0.2678	0.049*	
H14C	0.5047	0.4672	0.2382	0.049*	
C15	0.2467 (5)	0.48765 (15)	0.4726 (3)	0.0277 (8)	
H15A	0.1908	0.4986	0.5465	0.042*	
H15B	0.3564	0.4682	0.5063	0.042*	
H15C	0.1703	0.4618	0.4136	0.042*	
C16	0.1769 (5)	0.60788 (16)	0.5680 (3)	0.0291 (8)	
H16A	0.1548	0.5708	0.6062	0.044*	
H16B	0.0684	0.6296	0.5477	0.044*	
H16C	0.2615	0.6294	0.6312	0.044*	
C17	0.2342 (5)	0.70814 (14)	0.4072 (3)	0.0284 (8)	
H17A	0.3356	0.7331	0.4119	0.043*	
H17B	0.2020	0.7056	0.4945	0.043*	
H17C	0.1373	0.7241	0.3441	0.043*	
Cl1	−0.15438 (11)	0.63943 (4)	0.27606 (9)	0.0297 (2)	
Cl2	−0.08251 (10)	0.50113 (3)	0.18615 (8)	0.02257 (18)	
N	−0.0221 (4)	0.60308 (11)	0.0272 (3)	0.0209 (6)	
H1A	0.0225	0.5772	−0.0248	0.025*	
H1B	−0.1376	0.5945	0.0208	0.025*	
O	−0.1412 (4)	0.65784 (12)	−0.2709 (2)	0.0377 (6)	
Ru	0.10420 (3)	0.587116 (10)	0.23267 (2)	0.01582 (9)	

### Atomic displacement parameters (Å^2^)

**Table:**

	*U*^11^	*U*^22^	*U*^33^	*U*^12^	*U*^13^	*U*^23^
C1	0.071 (3)	0.032 (2)	0.0259 (19)	−0.008 (2)	−0.013 (2)	0.0090 (17)
C2	0.049 (3)	0.044 (3)	0.059 (3)	0.006 (2)	−0.027 (2)	0.011 (2)
C3	0.041 (2)	0.037 (2)	0.044 (2)	0.0089 (19)	0.0012 (19)	−0.0058 (18)
C4	0.0314 (19)	0.0203 (16)	0.0236 (16)	−0.0037 (15)	−0.0008 (14)	0.0029 (13)
C5	0.035 (2)	0.0264 (19)	0.0295 (18)	−0.0074 (16)	−0.0050 (15)	0.0062 (15)
C6	0.0107 (15)	0.0185 (15)	0.0259 (16)	−0.0042 (12)	−0.0011 (12)	−0.0005 (12)
C7	0.0136 (15)	0.0233 (16)	0.0241 (16)	−0.0020 (13)	0.0031 (12)	−0.0033 (13)
C8	0.0126 (15)	0.0195 (16)	0.0249 (16)	0.0029 (12)	0.0004 (12)	−0.0005 (13)
C9	0.0147 (16)	0.0207 (16)	0.0223 (15)	0.0001 (13)	−0.0027 (12)	0.0038 (12)
C10	0.0157 (15)	0.0257 (17)	0.0140 (14)	−0.0015 (13)	−0.0018 (12)	−0.0026 (12)
C11	0.0156 (16)	0.0200 (16)	0.0199 (15)	−0.0032 (13)	−0.0039 (12)	−0.0055 (12)
C12	0.0248 (18)	0.0246 (17)	0.0329 (18)	−0.0080 (15)	0.0039 (14)	0.0020 (14)
C13	0.0269 (18)	0.033 (2)	0.0263 (17)	−0.0018 (15)	0.0114 (14)	−0.0063 (14)
C14	0.031 (2)	0.0235 (18)	0.044 (2)	0.0068 (16)	0.0093 (17)	−0.0049 (15)
C15	0.0280 (19)	0.0251 (17)	0.0291 (18)	0.0007 (15)	0.0024 (15)	0.0070 (14)
C16	0.035 (2)	0.0317 (19)	0.0218 (16)	−0.0043 (16)	0.0071 (15)	−0.0031 (14)
C17	0.036 (2)	0.0207 (17)	0.0280 (17)	−0.0007 (15)	0.0035 (15)	−0.0072 (14)
Cl1	0.0201 (4)	0.0318 (5)	0.0365 (5)	0.0070 (4)	0.0036 (3)	−0.0094 (4)
Cl2	0.0227 (4)	0.0208 (4)	0.0239 (4)	−0.0054 (3)	0.0035 (3)	−0.0013 (3)
N	0.0230 (14)	0.0196 (14)	0.0188 (13)	0.0006 (11)	−0.0003 (11)	0.0025 (10)
O	0.0504 (17)	0.0359 (15)	0.0274 (13)	−0.0006 (13)	0.0083 (12)	0.0029 (11)
Ru	0.01421 (14)	0.01550 (14)	0.01747 (14)	0.00002 (10)	0.00214 (9)	−0.00114 (10)

### Geometric parameters (Å, °)

**Table:**

C1—C2	1.319 (7)	C10—Ru	2.209 (3)
C1—O	1.368 (5)	C11—C17	1.499 (4)
C1—H1	0.9300	C11—Ru	2.194 (3)
C2—C3	1.428 (6)	C12—H12A	0.9600
C2—H2	0.9300	C12—H12B	0.9600
C3—C4	1.338 (5)	C12—H12C	0.9600
C3—H3	0.9300	C13—H13A	0.9600
C4—O	1.360 (4)	C13—H13B	0.9600
C4—C5	1.478 (5)	C13—H13C	0.9600
C5—N	1.478 (4)	C14—H14A	0.9600
C5—H5A	0.9700	C14—H14B	0.9600
C5—H5B	0.9700	C14—H14C	0.9600
C6—C11	1.425 (4)	C15—H15A	0.9600
C6—C7	1.437 (4)	C15—H15B	0.9600
C6—C12	1.498 (4)	C15—H15C	0.9600
C6—Ru	2.211 (3)	C16—H16A	0.9600
C7—C8	1.412 (4)	C16—H16B	0.9600
C7—C13	1.505 (5)	C16—H16C	0.9600
C7—Ru	2.189 (3)	C17—H17A	0.9600
C8—C9	1.442 (4)	C17—H17B	0.9600
C8—C14	1.500 (4)	C17—H17C	0.9600
C8—Ru	2.184 (3)	Cl1—Ru	2.4277 (9)
C9—C10	1.415 (4)	Cl2—Ru	2.4299 (8)
C9—C15	1.507 (4)	N—Ru	2.156 (2)
C9—Ru	2.204 (3)	N—H1A	0.9000
C10—C11	1.437 (4)	N—H1B	0.9000
C10—C16	1.499 (4)		
			
C2—C1—O	110.0 (3)	C8—C14—H14B	109.5
C2—C1—H1	125.0	H14A—C14—H14B	109.5
O—C1—H1	125.0	C8—C14—H14C	109.5
C1—C2—C3	107.3 (4)	H14A—C14—H14C	109.5
C1—C2—H2	126.4	H14B—C14—H14C	109.5
C3—C2—H2	126.4	C9—C15—H15A	109.5
C4—C3—C2	105.9 (4)	C9—C15—H15B	109.5
C4—C3—H3	127.0	H15A—C15—H15B	109.5
C2—C3—H3	127.0	C9—C15—H15C	109.5
C3—C4—O	110.2 (3)	H15A—C15—H15C	109.5
C3—C4—C5	132.0 (4)	H15B—C15—H15C	109.5
O—C4—C5	117.8 (3)	C10—C16—H16A	109.5
N—C5—C4	114.4 (3)	C10—C16—H16B	109.5
N—C5—H5A	108.7	H16A—C16—H16B	109.5
C4—C5—H5A	108.7	C10—C16—H16C	109.5
N—C5—H5B	108.7	H16A—C16—H16C	109.5
C4—C5—H5B	108.7	H16B—C16—H16C	109.5
H5A—C5—H5B	107.6	C11—C17—H17A	109.5
C11—C6—C7	119.1 (3)	C11—C17—H17B	109.5
C11—C6—C12	120.5 (3)	H17A—C17—H17B	109.5
C7—C6—C12	120.3 (3)	C11—C17—H17C	109.5
C11—C6—Ru	70.48 (17)	H17A—C17—H17C	109.5
C7—C6—Ru	70.13 (17)	H17B—C17—H17C	109.5
C12—C6—Ru	134.3 (2)	C5—N—Ru	119.9 (2)
C8—C7—C6	120.3 (3)	C5—N—H1A	107.3
C8—C7—C13	119.4 (3)	Ru—N—H1A	107.3
C6—C7—C13	120.3 (3)	C5—N—H1B	107.3
C8—C7—Ru	70.96 (18)	Ru—N—H1B	107.3
C6—C7—Ru	71.76 (18)	H1A—N—H1B	106.9
C13—C7—Ru	130.4 (2)	C4—O—C1	106.6 (3)
C7—C8—C9	120.4 (3)	N—Ru—C8	118.85 (11)
C7—C8—C14	119.3 (3)	N—Ru—C7	96.38 (11)
C9—C8—C14	120.3 (3)	C8—Ru—C7	37.68 (12)
C7—C8—Ru	71.36 (18)	N—Ru—C11	125.70 (11)
C9—C8—Ru	71.56 (17)	C8—Ru—C11	80.97 (12)
C14—C8—Ru	129.9 (2)	C7—Ru—C11	68.50 (12)
C10—C9—C8	119.8 (3)	N—Ru—C9	155.51 (12)
C10—C9—C15	121.6 (3)	C8—Ru—C9	38.38 (12)
C8—C9—C15	118.5 (3)	C7—Ru—C9	68.63 (12)
C10—C9—Ru	71.52 (17)	C11—Ru—C9	68.20 (12)
C8—C9—Ru	70.07 (17)	N—Ru—C10	163.32 (11)
C15—C9—Ru	128.7 (2)	C8—Ru—C10	68.47 (11)
C9—C10—C11	119.6 (3)	C7—Ru—C10	81.08 (12)
C9—C10—C16	121.8 (3)	C11—Ru—C10	38.10 (11)
C11—C10—C16	118.5 (3)	C9—Ru—C10	37.40 (11)
C9—C10—Ru	71.08 (16)	N—Ru—C6	99.31 (11)
C11—C10—Ru	70.36 (16)	C8—Ru—C6	68.43 (12)
C16—C10—Ru	129.2 (2)	C7—Ru—C6	38.11 (11)
C6—C11—C10	120.8 (3)	C11—Ru—C6	37.75 (12)
C6—C11—C17	119.7 (3)	C9—Ru—C6	80.98 (12)
C10—C11—C17	119.4 (3)	C10—Ru—C6	68.52 (12)
C6—C11—Ru	71.77 (17)	N—Ru—Cl1	81.39 (8)
C10—C11—Ru	71.54 (17)	C8—Ru—Cl1	159.36 (9)
C17—C11—Ru	128.2 (2)	C7—Ru—Cl1	152.45 (9)
C6—C12—H12A	109.5	C11—Ru—Cl1	90.40 (9)
C6—C12—H12B	109.5	C9—Ru—Cl1	120.98 (9)
H12A—C12—H12B	109.5	C10—Ru—Cl1	93.22 (9)
C6—C12—H12C	109.5	C6—Ru—Cl1	114.82 (9)
H12A—C12—H12C	109.5	N—Ru—Cl2	78.72 (7)
H12B—C12—H12C	109.5	C8—Ru—Cl2	92.25 (8)
C7—C13—H13A	109.5	C7—Ru—Cl2	119.00 (8)
C7—C13—H13B	109.5	C11—Ru—Cl2	154.90 (9)
H13A—C13—H13B	109.5	C9—Ru—Cl2	91.51 (8)
C7—C13—H13C	109.5	C10—Ru—Cl2	117.02 (9)
H13A—C13—H13C	109.5	C6—Ru—Cl2	157.04 (9)
H13B—C13—H13C	109.5	Cl1—Ru—Cl2	87.68 (3)
C8—C14—H14A	109.5		
			
O—C1—C2—C3	−1.1 (5)	C8—C7—Ru—C10	66.14 (18)
C1—C2—C3—C4	1.0 (5)	C6—C7—Ru—C10	−66.39 (18)
C2—C3—C4—O	−0.6 (5)	C13—C7—Ru—C10	179.0 (3)
C2—C3—C4—C5	179.2 (4)	C8—C7—Ru—C6	132.5 (3)
C3—C4—C5—N	93.9 (5)	C13—C7—Ru—C6	−114.6 (4)
O—C4—C5—N	−86.4 (4)	C8—C7—Ru—Cl1	145.91 (17)
C11—C6—C7—C8	−1.2 (4)	C6—C7—Ru—Cl1	13.4 (3)
C12—C6—C7—C8	175.7 (3)	C13—C7—Ru—Cl1	−101.2 (3)
Ru—C6—C7—C8	−53.8 (3)	C8—C7—Ru—Cl2	−49.89 (19)
C11—C6—C7—C13	179.3 (3)	C6—C7—Ru—Cl2	177.58 (14)
C12—C6—C7—C13	−3.8 (4)	C13—C7—Ru—Cl2	63.0 (3)
Ru—C6—C7—C13	126.7 (3)	C6—C11—Ru—N	−52.9 (2)
C11—C6—C7—Ru	52.6 (2)	C10—C11—Ru—N	174.38 (17)
C12—C6—C7—Ru	−130.5 (3)	C17—C11—Ru—N	61.0 (3)
C6—C7—C8—C9	−0.1 (5)	C6—C11—Ru—C8	66.24 (19)
C13—C7—C8—C9	179.5 (3)	C10—C11—Ru—C8	−66.45 (19)
Ru—C7—C8—C9	−54.2 (3)	C17—C11—Ru—C8	−179.8 (3)
C6—C7—C8—C14	−179.8 (3)	C6—C11—Ru—C7	29.25 (18)
C13—C7—C8—C14	−0.3 (4)	C10—C11—Ru—C7	−103.4 (2)
Ru—C7—C8—C14	126.0 (3)	C17—C11—Ru—C7	143.2 (3)
C6—C7—C8—Ru	54.2 (3)	C6—C11—Ru—C9	103.9 (2)
C13—C7—C8—Ru	−126.3 (3)	C10—C11—Ru—C9	−28.76 (18)
C7—C8—C9—C10	0.7 (4)	C17—C11—Ru—C9	−142.1 (3)
C14—C8—C9—C10	−179.5 (3)	C6—C11—Ru—C10	132.7 (3)
Ru—C8—C9—C10	−53.4 (2)	C17—C11—Ru—C10	−113.4 (4)
C7—C8—C9—C15	178.2 (3)	C10—C11—Ru—C6	−132.7 (3)
C14—C8—C9—C15	−2.0 (4)	C17—C11—Ru—C6	113.9 (4)
Ru—C8—C9—C15	124.1 (3)	C6—C11—Ru—Cl1	−132.59 (17)
C7—C8—C9—Ru	54.1 (3)	C10—C11—Ru—Cl1	94.72 (17)
C14—C8—C9—Ru	−126.1 (3)	C17—C11—Ru—Cl1	−18.7 (3)
C8—C9—C10—C11	−0.1 (4)	C6—C11—Ru—Cl2	142.04 (19)
C15—C9—C10—C11	−177.5 (3)	C10—C11—Ru—Cl2	9.3 (3)
Ru—C9—C10—C11	−52.9 (2)	C17—C11—Ru—Cl2	−104.0 (3)
C8—C9—C10—C16	177.8 (3)	C10—C9—Ru—N	158.9 (2)
C15—C9—C10—C16	0.4 (5)	C8—C9—Ru—N	26.2 (3)
Ru—C9—C10—C16	125.1 (3)	C15—C9—Ru—N	−84.9 (4)
C8—C9—C10—Ru	52.7 (2)	C10—C9—Ru—C8	132.7 (3)
C15—C9—C10—Ru	−124.7 (3)	C15—C9—Ru—C8	−111.1 (4)
C7—C6—C11—C10	1.8 (4)	C10—C9—Ru—C7	103.8 (2)
C12—C6—C11—C10	−175.1 (3)	C8—C9—Ru—C7	−28.97 (17)
Ru—C6—C11—C10	54.3 (3)	C15—C9—Ru—C7	−140.1 (3)
C7—C6—C11—C17	−176.6 (3)	C10—C9—Ru—C11	29.27 (18)
C12—C6—C11—C17	6.5 (4)	C8—C9—Ru—C11	−103.5 (2)
Ru—C6—C11—C17	−124.2 (3)	C15—C9—Ru—C11	145.4 (3)
C7—C6—C11—Ru	−52.4 (3)	C8—C9—Ru—C10	−132.7 (3)
C12—C6—C11—Ru	130.7 (3)	C15—C9—Ru—C10	116.2 (4)
C9—C10—C11—C6	−1.2 (4)	C10—C9—Ru—C6	66.25 (19)
C16—C10—C11—C6	−179.1 (3)	C8—C9—Ru—C6	−66.48 (19)
Ru—C10—C11—C6	−54.4 (3)	C15—C9—Ru—C6	−177.6 (3)
C9—C10—C11—C17	177.3 (3)	C10—C9—Ru—Cl1	−47.4 (2)
C16—C10—C11—C17	−0.7 (4)	C8—C9—Ru—Cl1	179.92 (15)
Ru—C10—C11—C17	124.1 (3)	C15—C9—Ru—Cl1	68.8 (3)
C9—C10—C11—Ru	53.2 (2)	C10—C9—Ru—Cl2	−135.55 (17)
C16—C10—C11—Ru	−124.8 (3)	C8—C9—Ru—Cl2	91.72 (17)
C4—C5—N—Ru	−154.7 (3)	C15—C9—Ru—Cl2	−19.4 (3)
C3—C4—O—C1	−0.1 (4)	C9—C10—Ru—N	−148.7 (4)
C5—C4—O—C1	−179.9 (3)	C11—C10—Ru—N	−16.1 (5)
C2—C1—O—C4	0.7 (5)	C16—C10—Ru—N	95.2 (5)
C5—N—Ru—C8	−106.9 (3)	C9—C10—Ru—C8	−29.36 (18)
C5—N—Ru—C7	−74.8 (3)	C11—C10—Ru—C8	103.3 (2)
C5—N—Ru—C11	−6.8 (3)	C16—C10—Ru—C8	−145.4 (3)
C5—N—Ru—C9	−125.1 (3)	C9—C10—Ru—C7	−66.30 (19)
C5—N—Ru—C10	5.4 (5)	C11—C10—Ru—C7	66.35 (19)
C5—N—Ru—C6	−36.4 (3)	C16—C10—Ru—C7	177.7 (3)
C5—N—Ru—Cl1	77.5 (3)	C9—C10—Ru—C11	−132.6 (3)
C5—N—Ru—Cl2	166.8 (3)	C16—C10—Ru—C11	111.3 (4)
C7—C8—Ru—N	59.6 (2)	C11—C10—Ru—C9	132.6 (3)
C9—C8—Ru—N	−167.94 (16)	C16—C10—Ru—C9	−116.0 (4)
C14—C8—Ru—N	−53.5 (3)	C9—C10—Ru—C6	−103.7 (2)
C9—C8—Ru—C7	132.4 (3)	C11—C10—Ru—C6	28.92 (18)
C14—C8—Ru—C7	−113.1 (4)	C16—C10—Ru—C6	140.2 (3)
C7—C8—Ru—C11	−66.33 (18)	C9—C10—Ru—Cl1	140.83 (17)
C9—C8—Ru—C11	66.11 (19)	C11—C10—Ru—Cl1	−86.53 (18)
C14—C8—Ru—C11	−179.4 (3)	C16—C10—Ru—Cl1	24.8 (3)
C7—C8—Ru—C9	−132.4 (3)	C9—C10—Ru—Cl2	51.8 (2)
C14—C8—Ru—C9	114.5 (4)	C11—C10—Ru—Cl2	−175.56 (15)
C7—C8—Ru—C10	−103.8 (2)	C16—C10—Ru—Cl2	−64.3 (3)
C9—C8—Ru—C10	28.66 (17)	C11—C6—Ru—N	138.96 (18)
C14—C8—Ru—C10	143.1 (3)	C7—C6—Ru—N	−88.47 (18)
C7—C8—Ru—C6	−29.28 (17)	C12—C6—Ru—N	25.0 (3)
C9—C8—Ru—C6	103.2 (2)	C11—C6—Ru—C8	−103.6 (2)
C14—C8—Ru—C6	−142.4 (3)	C7—C6—Ru—C8	28.97 (18)
C7—C8—Ru—Cl1	−132.6 (2)	C12—C6—Ru—C8	142.4 (4)
C9—C8—Ru—Cl1	−0.2 (4)	C11—C6—Ru—C7	−132.6 (3)
C14—C8—Ru—Cl1	114.3 (3)	C12—C6—Ru—C7	113.4 (4)
C7—C8—Ru—Cl2	137.98 (17)	C7—C6—Ru—C11	132.6 (3)
C9—C8—Ru—Cl2	−89.59 (17)	C12—C6—Ru—C11	−114.0 (4)
C14—C8—Ru—Cl2	24.9 (3)	C11—C6—Ru—C9	−65.85 (19)
C8—C7—Ru—N	−130.50 (18)	C7—C6—Ru—C9	66.72 (19)
C6—C7—Ru—N	96.97 (18)	C12—C6—Ru—C9	−179.8 (3)
C13—C7—Ru—N	−17.6 (3)	C11—C6—Ru—C10	−29.17 (18)
C6—C7—Ru—C8	−132.5 (3)	C7—C6—Ru—C10	103.4 (2)
C13—C7—Ru—C8	112.9 (4)	C12—C6—Ru—C10	−143.2 (4)
C8—C7—Ru—C11	103.5 (2)	C11—C6—Ru—Cl1	54.20 (19)
C6—C7—Ru—C11	−28.98 (18)	C7—C6—Ru—Cl1	−173.23 (15)
C13—C7—Ru—C11	−143.6 (3)	C12—C6—Ru—Cl1	−59.8 (3)
C8—C7—Ru—C9	29.47 (18)	C11—C6—Ru—Cl2	−138.00 (19)
C6—C7—Ru—C9	−103.05 (19)	C7—C6—Ru—Cl2	−5.4 (3)
C13—C7—Ru—C9	142.3 (3)	C12—C6—Ru—Cl2	108.0 (3)

### Hydrogen-bond geometry (Å, °)

**Table:**

*D*—H···*A*	*D*—H	H···*A*	*D*···*A*	*D*—H···*A*
N—H1A···Cl2^i^	0.9	2.52	3.406 (3)	168.

Symmetry codes: (i) −*x*, −*y*+1, −*z*.
